# QM7-X, a comprehensive dataset of quantum-mechanical properties spanning the chemical space of small organic molecules

**DOI:** 10.1038/s41597-021-00812-2

**Published:** 2021-02-02

**Authors:** Johannes Hoja, Leonardo Medrano Sandonas, Brian G. Ernst, Alvaro Vazquez-Mayagoitia, Robert A. DiStasio Jr., Alexandre Tkatchenko

**Affiliations:** 1 Department of Physics and Materials Science, University of Luxembourg, L-1511 Luxembourg City, Luxembourg; 2 Institute of Chemistry, University of Graz, 8010 Graz, Austria; 3 Department of Chemistry and Chemical Biology, Cornell University, Ithaca, NY 14853 USA; 4 Computational Science Division, Argonne National Laboratory, Lemont, IL 60439 USA

**Keywords:** Computational chemistry, Chemical physics, Cheminformatics

## Abstract

We introduce QM7-X, a comprehensive dataset of 42 physicochemical properties for ≈4.2 million equilibrium and non-equilibrium structures of small organic molecules with up to seven non-hydrogen (C, N, O, S, Cl) atoms. To span this fundamentally important region of chemical compound space (CCS), QM7-X includes an exhaustive sampling of (meta-)stable equilibrium structures—comprised of constitutional/structural isomers and stereoisomers, e.g., enantiomers and diastereomers (including *cis-*/*trans*- and conformational isomers)—as well as 100 non-equilibrium structural variations thereof to reach a total of ≈4.2 million molecular structures. Computed at the tightly converged quantum-mechanical PBE0+MBD level of theory, QM7-X contains global (molecular) and local (atom-in-a-molecule) properties ranging from ground state quantities (such as atomization energies and dipole moments) to response quantities (such as polarizability tensors and dispersion coefficients). By providing a systematic, extensive, and tightly-converged dataset of quantum-mechanically computed physicochemical properties, we expect that QM7-X will play a critical role in the development of next-generation machine-learning based models for exploring greater swaths of CCS and performing in silico design of molecules with targeted properties.

## Abstract (summary)

**Table:** 

Measurement(s)	Chemical Properties • Physical Properties
Technology Type(s)	quantum chemistry computational method
Factor Type(s)	molecule

Machine-accessible metadata file describing the reported data: 10.6084/m9.figshare.13424984

## Background & Summary

A crucial aspect of drug discovery^1^ and molecular materials design^2^ is an extensive exploration and understanding of chemical compound space (CCS)—the extremely high-dimensional space containing all feasible molecular compositions and conformations. Recently, the combination of quantum mechanical (QM) calculations with machine learning (ML) has led to considerable insight into CCS^3–9^. However, progress along these lines can only happen with the availability of extensive and comprehensive QM-based datasets that adequately describe the complex structure–property relationships in molecules across CCS. In this regard, one challenge that is encountered during the generation of such datasets is the fact that their dimension scales exponentially with molecule size, thereby making it difficult to explore increasingly large swaths of CCS. The second challenge is the steep computational cost of tightly converged QM calculations, which are critical for obtaining an accurate and reliable description of the structure and physicochemical properties of each molecule.

To begin such an extensive exploration of CCS, the GDB datasets^1,10,11^ have enumerated up to 166 B organic molecules containing up to 17 heavy (non-hydrogen) atoms. Encoded as canonical SMILES (simplified molecular-input line-entry system) strings, the GDB datasets only provide the molecular formula and chemical connectivity, and do not contain any structural or molecular property information. As such, QM calculations on small subsets of the GDB datasets have subsequently been used to generate meta-stable conformations for each molecular composition. This has led to seminal QM-based datasets like QM7^10,12,13^ and QM9^11,14^, which are comprised of a single meta-stable molecular structure per SMILES string with up to seven and nine heavy (non-hydrogen) atoms, respectively. The QM7 and QM9 datasets have been widely used for benchmarking ML approaches and exploring molecular structure–property correlations^3^. In addition, molecular dynamics (MD) based datasets have become available for a few selected molecules and solids, and contain both equilibrium and non-equilibrium structures^15–21^; such datasets are becoming increasingly more useful for constructing advanced interatomic potentials^16,22,23^.

A more substantial coverage of CCS for small organic molecules was provided by Smith *et al*.^24,25^ with the generation of the ANI-1 dataset, which consists of more than 20 M equilibrium and non-equilibrium conformations of molecules containing up to eight heavy (C, N, O) atoms from GDB-11^26,27^. More recently, the ANI-1x dataset^28^ was introduced, which also contains 20 physicochemical properties for about 5 M structures computed using the semi-empirical *ω* B97X^29^ density functional. In addition, Smith *et al*.^28^ also provided local CCSD(T) energies for a smaller subset of 0.5 M structures. To date, the ANI-1 datasets contain the largest available collection of GDB-based QM calculations of molecular structures and properties. However, four challenges still remain to enable a systematic exploration of CCS for small organic molecules: (i) providing a systematic sampling of CCS in terms of constitutional/structural isomers and stereoisomers (*e.g*., enantiomers and diastereomers, including *cis-/trans-* and meta-stable conformational isomers), (ii) assessing the accuracy and reliability of QM structures and properties with respect to the employed density-functional approximation (especially for non-equilibrium conformations), (iii) offering a large set of local (atom-in-a-molecule) and global (molecular) physicochemical properties that would enable a comprehensive exploration of structure–property relationships throughout CCS, and (iv) providing accurate and reliable QM data that will enable the construction of models for describing covalent and non-covalent van der Waals (vdW) interactions.

**Fig. 1:**
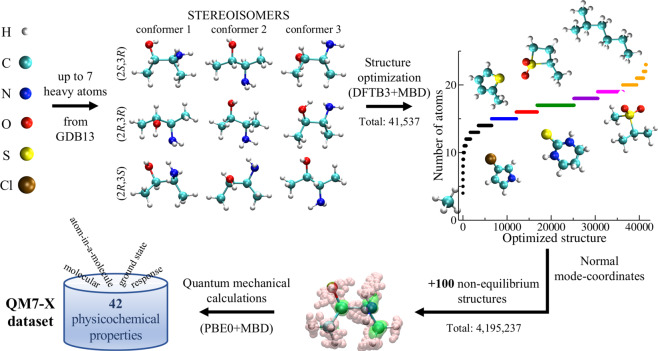
Schematic representation of the QM7-X dataset. The “building blocks” for the QM7-X dataset are the set of ≈7 k molecular formulae which contain up to seven heavy (non-hydrogen) atoms from the GDB13 database. The first step in the construction of QM7-X is an extensive sampling of the structural/constitutional isomers and stereoisomers, *e.g*., enantiomers and diastereomers (including *cis-/trans-* and conformational isomers) for each of the initial ≈7 k molecular formulae using the MMFF94 force field (schematically depicted above for some of the stereoisomers of 3-Amino-2-butanol). Each of these molecules was optimized at the DFTB3+MBD level of theory for a total of ≈40 k (meta-)stable equilibrium molecular structures. For each of these equilibrium structures, 100 non-equilibrium structures were generated by displacing the atoms according to linear combinations of the DFTB3+MBD normal modes for a total of ≈4.2 M molecular structures (with the range of atomic displacements (hydrogen/non-hydrogen atoms in pink/green) depicted above for a single 3-Amino-2-butanol conformer). We then performed QM calculations at the PBE0+MBD level to obtain 42 physicochemical properties for each of the ≈4.2 M molecular structures.

In order to convincingly address these four challenges in this work, we present QM7-X, which aims to provide a systematic, extensive, and tightly converged dataset of QM-based physical and chemical properties for a fundamentally important region of CCS covering small organic molecules (see Fig. 1). To do so, we performed a systematic and exhaustive sampling of the (meta-)stable equilibrium structures of all molecules with up to seven heavy (C, N, O, S, Cl) atoms in the GDB13 database^10^ using a density-functional tight binding (DFTB) approach; this includes constitutional/structural isomers and stereoisomers, *e.g*., enantiomers and diastereomers (including *cis-/trans-* and conformational isomers). This was followed by the generation of 100 non-equilibrium structures (*via* DFTB normal-mode displacements of each equilibrium structure) for a total of ≈4.2 M molecular structures. For each of these equilibrium and non-equilibrium molecular structures, QM7-X also includes an extensive number of QM-obtained physicochemical properties, most of which were computed using non-empirical hybrid density-functional theory (DFT) with a many-body treatment of vdW dispersion interactions (*i.e*., PBE0+MBD) in conjunction with tightly-converged numeric atom-centered orbitals^30^. In total, QM7-X contains 42 molecular (global) and atom-in-a-molecule (local) properties, which range from ground state quantities (such as total and atomization energies, atomic forces, HOMO-LUMO gaps, dipole/quadrupole moments, Hirshfeld quantities, etc.) to response quantities (such as polarizability tensors and dispersion coefficients)—all of which could be utilized for the construction of next-generation intra- and inter-molecular force fields. As such, we expect that QM7-X will be useful for the development of accurate and reliable ML-based techniques that will provide new insight into the complex structure–property relationships in molecules, and ultimately allow for more extensive exploration of CCS and the rational design of molecules with tailored properties.

## Methods

### Generation of equilibrium structures

As a basis for the QM7-X dataset, we considered all molecules containing up to seven heavy (non-hydrogen) atoms in the GDB13 database^10^, which provides an enumeration of the CCS spanned by small organic molecules comprised of H, C, N, O, S, and Cl atoms. These molecules range in size from *N* = 4–23 atoms (see Fig. 1). For each molecular formula, the GDB13 database only includes chemical connectivity information (*i.e*., some structural/constitutional isomers) encoded as SMILES strings. To generate *all* of the corresponding structural/constitutional isomers and stereoisomers, *e.g*., enantiomers and diastereomers (including *cis-/trans-* isomers) for each molecular formula, we created canonical SMILES strings for each possible molecular structure. Based on these SMILES strings, initial 3D structures were obtained using the MMFF94 force field^31–35^ *via* the gen3d option in Open Babel^36^. For each of these structures, we also generated a set of sufficiently different (meta-)stable conformational isomers, *i.e*., isomers which can be interconverted by rotations around single bonds. To do this, we performed a conformational isomer search with the Confab tool^37^ in conjunction with the MMFF94 force field. At the MMFF94 level, we retained the set of conformers that were within 50 kcal/mol of the most stable structure and differed by a root-mean-square deviation (RMSD) of 0.5 Å.

All molecular structures were subsequently re-optimized with third-order self-consistent charge density functional tight binding (DFTB3)^38–40^ supplemented with a treatment of many-body dispersion (MBD) interactions^41–44^, using the 3ob parameters^45,46^. All DFTB3+MBD calculations were performed using in-house versions of the DFTB+ code^47^ and the Atomic Simulation Environment (ASE)^48^. The lowest-energy structure obtained at the DFTB3+MBD level was considered the first conformer, and additional structures were accepted as separate conformers if the RMSD between the respective structure and the first conformer (as well as all subsequently accepted conformers) was larger than 1.0 Å. While most of these molecular structures correspond to local minima at the DFTB3+MBD level, we note in passing that some correspond to saddle points on the DFTB3+MBD potential energy surface (PES).

### Generation of non-equilibrium structures

In order to sample some of the non-equilibrium sectors of CCS, we generated 100 non-equilibrium structures for each of the (meta-)stable equilibrium structures described above. This was achieved by displacing each molecular structure along a linear combination of normal mode coordinates computed at the DFTB3+MBD level within the harmonic approximation (for consistency with the level of theory used to optimize the corresponding molecular structures). In order to generate comparable displacements for all equilibrium molecular structures (despite differing molecular sizes and relative stabilities), we generated a set of displaced structures which has an *average* energy difference (with respect to the corresponding (meta-)stable equilibrium structure) of

1 $$\left\langle \Delta E\right\rangle =\frac{3}{2}N{k}_{B}T,$$

in analogy to the equipartition theorem from classical statistical mechanics, in which each of the 3*N* degrees of freedom in a monatomic ideal gas contributes $\frac{1}{2}{k}_{B}T$ to the internal energy (with *k*_*B*_ being the Boltzmann constant and *T* the temperature). To sufficiently sample the PES for each (meta-)stable equilibrium structure, we set *T* = 1500 K. In addition to the <Δ*E>* convention defined in Eq. (1), the 100 non-equilibrium molecular structures were also required to follow the corresponding Boltzmann distribution:

2 $$P(\Delta E)=exp\left[-\frac{\Delta E}{{k}_{B}T}\right].$$

To generate a set of *candidate* non-equilibrium molecular structures, we randomly chose an energy difference (Δ*ε*) for each of the 3*N*–6 (or 3*N*–5 in the case of a diatomic and/or linear polyatomic molecule) vibrational/normal modes in a given molecule. For the *i*-th vibrational/normal mode (with frequency *v*_*i*_), the corresponding displacement amplitude was computed *via* ${A}_{i}=\frac{\sqrt{2\,\Delta {\varepsilon }_{i}}}{{\nu }_{i}}$, and the corresponding displacement vector (along this mode) was obtained by multiplying the eigenvectors with *A*_*i*_ and a randomized sign. A candidate non-equilibrium molecular structure was then obtained by simultaneously perturbing the equilibrium molecular structure along every displacement vector. Since the generation of candidate non-equilibrium molecular structures *via* a linear combination of normal-mode displacements assumes complete independence of the normal modes as well as the validity of the harmonic approximation, the actual Δ*E* corresponding to each candidate was explicitly calculated with DFTB3+MBD and the required Boltzmann distribution in Eq. (2) was strictly enforced. To do so, we pre-defined a histogram that accounts for 100 structures and covers a range of Δ*E* values from 0.0 to $4.5\times \frac{3}{2}N{k}_{B}T$, as shown in Fig. S1 of the Supplementary Information (SI). To generate a final set of 100 non-equilibrium molecular structures which meets the aforementioned criteria, the corresponding distribution of Δ*E* values had to fit this histogram. More specifically, we accepted candidate structures (with an RMSD >0.0075*N* Å to all previously accepted structures) until all of the histogram bins were filled.

The approximately 4.2 M equilibrium and non-equilibrium structures generated using these procedures form the QM7-X dataset (see Table 1). Since we were unable to obtain a complete energy distribution (as described above) for 261 of the 7,211 molecular formulae taken from the GDB13 dataset, molecules with these molecular formulae were not included in QM7-X. As such, QM7-X covers 96.4% of all molecules containing up to seven heavy atoms from GDB13 (and 98.8% of the corresponding (meta-)stable stereoisomers), which should provide a sufficiently accurate and representative sample of the small organic molecules contained in CCS. We note in passing that some of the generated equilibrium structures are not entirely unique, *e.g*., some of the stereoisomers of molecules with multiple chiral centers were identical as well as some of the (rotational) conformational isomers; permutations of identical atoms were not initially considered when computing RMSD values utilizing the RMSD minimization tool available in ASE^49^. Therefore, approximately 4.6% of the equilibrium structures constitute duplicates (see file DupMols.dat in the ZENODO repository^50^), which were identified by an additional *a posteriori* approach that considered similarities between potential duplicate structures in the following six physicochemical properties: PBE0 energy, MBD energy, HOMO-LUMO gap, HOMO energy, molecular polarizability, and total dipole moment. The threshold used to check for similarity was set to 10^−3^ × the corresponding unit for each property. While the presence of some duplicate equilibrium structures in the QM7-X dataset might influence the performance of ML models developed using this data, the non-equilibrium structures associated with these duplicate equilibrium structures are **not identical** and probe different regions of the molecular PES. As such, their inclusion in QM7-X covers a larger swath of molecular property space, and can contribute to the development of more robust ML models for predicting the physicochemical properties in the QM7-X dataset. Thus, we offer users the option to keep or exclude these duplicate equilibrium structures (as well as their corresponding non-equilibrium structures) in the QM7-X dataset *via* the createDB.py script uploaded to the ZENODO repository^50^.

**Table 1:** Content and size of the QM7-X dataset.

Heavy Atoms	Molecules from GDB13	Stereoisomers	Equilibrium Structures	Total Structures (Equilibrium + Non-Equilibrium)
1	1	1	1	101
2	2	2	2	202
3	10	10	10	1,010
4	42	45	58	5,858
5	149	193	351	35,451
6	901	1,400	3,677	371,377
7	5,845	11,627	37,438	3,781,238
Total	6,950	13,278	41,537	4,195,237

The number of molecules from GDB13, stereoisomers (*e.g*., enantiomers and *cis-/trans-* diastereomers), (meta-)stable equilibrium structures (including conformational isomers), and the total number of equilibrium and non-equilibrium structures are listed for different numbers of heavy atoms and the entire QM7-X dataset.

### Calculation of physicochemical properties

These ≈4.2 M DFTB3+MBD structures were now utilized for more accurate QM single-point calculations using dispersion-inclusive hybrid DFT. Energies, forces, and several other physicochemical properties (see Table 2) were calculated at the PBE0+MBD^41,51,52^ level using the FHI-aims code^53,54^ (version 180218). For all calculations, “tight” settings were applied for basis functions and integration grids. Energies were converged to 10^−6^ eV and the accuracy of the forces was set to 10^−4^ eV/Å. The convergence criteria used during self-consistent field (SCF) optimizations were 10^−3^ eV for the sum of eigenvalues and 10^−6^ electrons/Å^3^ for the charge density.

**Table 2:** List of physicochemical properties in the QM7-X dataset.

#	Symbol	Property	Unit	Dimension	Type	Level	HDF5 keys
1	*Z*	Atomic numbers	—	*N*	S	—	‘atNUM’
2	*R*	Atomic positions (coordinates)	Å	3 *N*	S	TB	‘atXYZ’
3	Δ*R*	RMSD to optimized structure	Å	1	S	—	‘sRMSD’
4	*I*	Moment of inertia tensor	amu·Å^2^	9	S	—	‘sMIT’
5	*E*_tot_	Total PBE0+MBD energy	eV	1	M,G	P0M	‘ePBE0+MBD’
6	*E*_TB_	Total DFTB3+MBD energy	eV	1	M,G	TB	‘eDFTB+MBD’
7	*E*_at_	Atomization energy	eV	1	M,G	P0	‘eAT’
8	*E*_PBE0_	PBE0 energy	eV	1	M,G	P0	‘ePBE0’
9	*E*_MBD_	MBD energy	eV	1	M,G	P0M	‘eMBD’
10	*E*_TS_	TS dispersion energy	eV	1	M,G	P0	‘eTS’
11	*E*_nn_	Nuclear-nuclear repulsion energy	eV	1	M,G	—	‘eNN’
12	*E*_kin_	Kinetic energy	eV	1	M,G	P0	‘eKIN’
13	*E*_ne_	Nuclear-electron attraction	eV	1	M,G	P0	‘eNE’
14	*E*_coul_	Classical coulomb energy (el-el)	eV	1	M,G	P0	‘eEE’
15	*E*_xc_	Exchange-correlation energy	eV	1	M,G	P0	‘eXC’
16	*E*_x_	Exchange energy	eV	1	M,G	P0	‘eX’
17	*E*_c_	Correlation energy	eV	1	M,G	P0	‘eC’
18	*E*_xx_	Exact exchange energy	eV	1	M,G	P0	‘eXX’
19	*E*_KS_	Sum of Kohn-Sham eigenvalues	eV	1	M,G	P0	’eKSE’
20	*ε*	Kohn-Sham eigenvalues	eV	*	M,G	P0	‘KSE’
21	*E*_HOMO_	HOMO energy	eV	1	M,G	P0	‘eH’
22	*E*_LUMO_	LUMO energy	eV	1	M,G	P0	‘eL’
23	*E*_gap_	HOMO-LUMO gap	eV	1	M,G	P0	‘HLgap’
24	*D*_s_	Scalar dipole moment	*e·*Å	1	M,G	P0	‘DIP’
25	*D*	Dipole moment	*e·*Å	3	M,G	P0	‘vDIP’
26	*Q*_tot_	Total quadrupole moment	*e·*Å^2^	3	M,G	P0	‘vTQ’
27	*Q*_ion_	Ionic quadrupole moment	*e·*Å^2^	3	M,G	P0	‘vIQ’
28	*Q*_elec_	Electronic quadrupole moment	*e·*Å^2^	3	M,G	P0	‘vEQ’
29	*C*_6_	Molecular *C*_6_ coefficient	${E}_{h}\cdot {a}_{0}^{3}$	1	M,R	P0M	‘mC6’
30	*α*_s_	Molecular polarizability (isotropic)	${a}_{0}^{3}$	1	M,R	P0M	‘mPOL’
31	*α*	Molecular polarizability tensor	${a}_{0}^{3}$	9	M,R	P0M	‘mTPOL’
32	*F*_tot_	Total PBE0+MBD atomic forces	eV/Å	3 *N*	A,G	P0M	‘totFOR’
33	*F*_PBE0_	PBE0 atomic forces	eV/Å	3 *N*	A,G	P0	’pbe0FOR’
34	*F*_MBD_	MBD atomic forces	eV/Å	3 *N*	A,G	P0M	‘vdwFOR’
35	*V*_H_	Hirshfeld volumes	${a}_{0}^{3}$	*N*	A,G	P0	‘hVOL’
36	*V*_ratio_	Hirshfeld ratios	—	*N*	A,G	P0	‘hRAT’
37	*q*_H_	Hirshfeld charges	*e*	*N*	A,G	P0	‘hCHG’
38	*D*_H,s_	Scalar Hirshfeld dipole moments	*e·a*_0_	*N*	A,G	P0	‘hDIP’
39	*D*_H_	Hirshfeld dipole moments	*e·a*_0_	3 *N*	A,G	P0	‘hVDIP’
40	${\widetilde{C}}_{6}$	Atomic ${C}_{6}$ coefficients	${E}_{h}\cdot {a}_{0}^{6}$	*N*	A,R	P0M	‘atC6’
41	${\widetilde{\alpha }}_{s}$	Atomic polarizabilities (isotropic)	${a}_{0}^{3}$	*N*	A,R	P0M	‘atPOL’
42	*R*_vdW_	vdW radii	*a*_0_	*N*	A,R	P0M	‘vdwR’

Each property is represented by a symbol (with units and dimension) and can be found in the HDF5 files using the corresponding HDF5 keys. Different property types are distinguished as follows: structural (S), molecular (M), atom-in-a-molecule (A), ground-state (G), and response (R). Different levels of theory are indicated as follows: DFTB3+MBD (TB), PBE0 (P0), and PBE0+MBD (P0M). The P0M label indicates which properties explicitly include dispersion interactions. ${E}_{h}$ and ${a}_{0}$ refer to the atomic units of energy (Hartree) and length (Bohr radius), respectively.

*The number of Kohn-Sham eigenvalues varies for each molecule.

Here, the MBD energies, MBD atomic forces, atomic *C*_6_ coefficients, and isotropic atomic polarizabilities were computed using the range-separated self-consistent screening (rsSCS) approach^42^, while the molecular *C*_6_ coefficients and polarizabilities (both isotropic and tensor) were obtained *via* the SCS approach^41^. Hirshfeld ratios correspond to the Hirshfeld volumes divided by the free atom volumes. The TS dispersion energy refers to the pairwise Tkatchenko-Scheffler (TS) dispersion energy in conjunction with the PBE0 functional^55^. The vdW radii were also obtained using the SCS approach *via* ${R}_{{\rm{vdW}}}={\left({\alpha }^{{\rm{SCS}}}/{\alpha }^{{\rm{TS}}}\right)}^{1/3}{R}_{{\rm{vdW}}}^{{\rm{TS}}}$, where *α*^TS^ and ${R}_{{\rm{v}}{\rm{d}}{\rm{W}}}^{{\rm{T}}{\rm{S}}}$ are the atomic polarizability and vdW radius computed according to the TS scheme, respectively. Atomization energies were obtained by subtracting the atomic PBE0 energies from the PBE0 energy of each molecular conformation (see Table S2). The exact exchange energy is the amount of exact (or Hartree-Fock) exchange that has been admixed into the exchange-correlation energy.

## Data Records

The QM7-X dataset is provided in eight HDF5 based files in a ZENODO.ORG data repository^50^. One can also find there a README file with technical usage details and examples of how to access the information stored in QM7-X (with and without considering duplicates, see createDB.py file).

### HDF5 file format

The information for each molecular structure is stored in a Python dictionary (dict) type containing all relevant properties and recorded in *groups* in HDF5 file format. HDF5 keys to access the atomic numbers, atomic positions (coordinates), and physicochemical properties in each dictionary are provided in Table 2. The dimension of each array depends on the number of atoms and the required property, *e.g*., for a methane (CH_4_) molecule ‘atNUM’ is a 1D array of *N* = 5 elements ([6, 1, 1, 1, 1]) while ‘atXYZ’ is a 2D array comprised of *N* = 5 rows and three columns (*x*, *y*, *z* coordinates). All structures are labeled as Geom-mr-is-ct-u, where r enumerates the SMILES strings, s the stereoisomers (excluding conformational isomers), *t* the considered (meta-)stable conformational isomers, and *u* the optimized/displaced structures (*u* = opt indicates the DFTB3+MBD optimized structures and *u* = 1, …, 100 indicates the displaced non-equilibrium structures). We note in passing that the indices used in the QM7-X dataset reflect the order in which a given structure was generated and do not correspond to sorted DFTB3+MBD (or PBE0+MBD) energies.

## Technical Validation

In contrast to many other QM-based datasets like QM7 and QM9, which contain a single structure per molecule, the QM7-X dataset not only includes constitutional/structural isomers, but also stereoisomers (*e.g*., enantiomers and diastereomers, including *cis-/trans-* and conformational isomers) as well as non-equilibrium structural variations thereof. While many of the considered molecules do overlap with those in the ANI-1 dataset^24^, QM7-X also contains a more extensive sampling of equilibrium stereoisomers and a significantly different sampling of non-equilibrium structures (*vide infra*). Moreover, the number of reported physicochemical properties and the employed level of electronic structure theory in QM7-X is substantially more advanced compared to prior work. Therefore, the QM7-X dataset contains significantly more data for flexible molecules with stereocenters than for rigid molecules without stereocenters. Since stereochemistry can play an important role in drug design, we consider that the data provided in this work will enable ML models to capture the subtle physicochemical differences existing between stereoisomers.

**Fig. 2:**
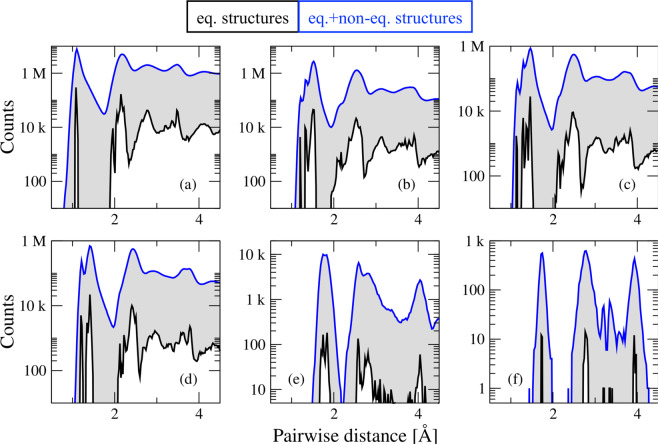
Distribution of atom-pairwise distances in the QM7-X dataset. Corresponding atom-pair distance distributions for: (**a**) C-H, (**b**) C-C, (**c**) C-N, (**d**) C-O, (**e**) C-S, and (**f**) C-Cl in the QM7-X dataset. For each distribution, we show the results corresponding to the equilibrium structures only (black lines) as well as the entire QM7-X dataset (blue lines). Contributions from non-equilibrium structures are highlighted in grey.

Another important target of the QM7-X dataset is efficient PES sampling for a large number of small organic molecules. Since the most crucial regions of the PES are reasonably close to the relevant (meta-)stable equilibrium structures, QM7-X contains 100 distorted/non-equilibrium structures for every such conformer. While most of these structures are in the vicinity of the optimized/equilibrium structures, some correspond to more highly distorted structures on the PES. Since it was only computationally feasible to consider 100 non-equilibrium structures at the PBE0+MBD level, these structures were chosen to cover as much of the PES surrounding each (meta-)stable equilibrium structure as possible. As such, we created these non-equilibrium structures by displacing the atoms in a given molecule along linear combinations of normal modes. Although a similar approach was utilized during the construction of the ANI-1 dataset^24^, we have eliminated all issues related to the inaccuracy of the harmonic approximation by recomputing the energy of every candidate structure and only accepting those structures with a Boltzmann energy distribution (see Methods). In doing so, we ensure that the relevant regions of each PES are well-sampled and only a small (and pre-determined) number of high-energy non-equilibrium structures are included in our dataset. The inclusion of non-equilibrium structures for each (meta-)stable equilibrium structure also provides better coverage of the conformational space than initially provided by the equilibrium structures, as seen in the pairwise distance distribution plots shown in Fig. 2.

In this work, the structure generation process was performed using DFTB3+MBD, while the subsequent energy, force, and property calculations were performed at the more robust PBE0+MBD level of theory. Since the focus of this work is the physicochemical properties of non-equilibrium structures surrounding (meta-)stable equilibrium points on each PES, the use of these two methods does not introduce any significant complications and/or errors. Although the PBE0+MBD (relative) energies will not strictly follow the same Boltzmann distribution as that used to generate the molecular structures at the DFTB3+MBD level, these two distributions are often very similar and lead to virtually the same <Δ*E>*. An average over all histograms is depicted in Fig. S1, where one can see some minor variations in the heights of the first few bins and only an insignificant amount of structures located slightly outside of the initially envisioned window of Δ*E* values. In addition, we have also quantitatively analyzed the structural agreement between conformers optimized at the DFTB3+MBD and PBE0+MBD levels. To do this, the DFTB3+MBD-optimized structures of 10 flexible molecules with at least five (meta-)stable conformers (see SI for more details) were randomly selected and re-optimized with PBE0+MBD, followed by a (harmonic) vibrational frequency analysis at the same level of theory. In doing so, it was found that all 63 so obtained PBE0+MBD geometries constitute local or global minima (*i.e*., with no imaginary frequencies), and the average RMSD between the DFTB3+MBD and PBE0+MBD structures amounts to only 0.1 Å. These structural differences are visualized for several conformers in Figs. S3–S5 in the SI. Even for the conformer with the largest observed RMSD of 0.66 Å, we note that the PBE0+MBD structure did not move to any of the other considered conformers, and resulted from modifications to the backbone structure. From this analysis, we would argue that the DFTB3+MBD-optimized structures are in excellent agreement with those optimized at the PBE0+MBD level, and hence provide a high-fidelity representation of the critical points on the PBE0+MBD PES.

**Fig. 3:**
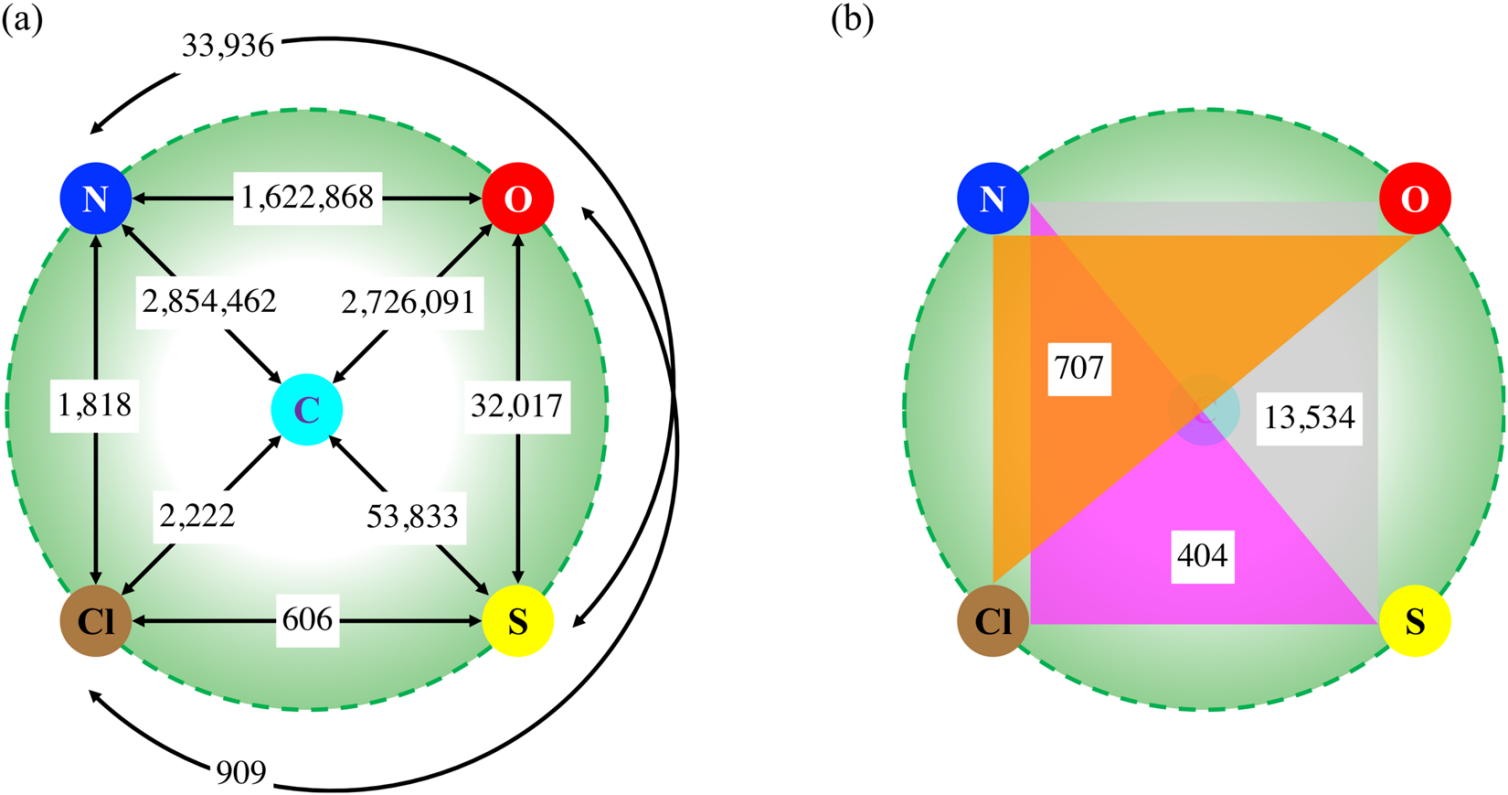
Statistical analysis of the molecular composition of the QM7-X dataset. Schematic illustrations representing the number of structures containing at least (**a**) two or (**b**) three different heavy (non-hydrogen) elements. Note that all molecules in QM7-X contain C and H atoms. Triple combinations including C atoms are not shown in (**b**) since that information can be extracted from (**a**).

In QM7-X, molecules made of seven heavy atoms are the most abundant with 37,438 equilibrium structures (including conformational isomers) and 3,743,800 non-equilibrium structures, followed by molecules containing six heavy atoms (see Table 1). C_5_H_11_NO turned out to be the most plentiful molecular formula with 3,200 (meta-)stable equilibrium structures (see Table S1). To examine the molecular composition of the QM7-X dataset, we have performed a statistical analysis by counting the number of structures containing at least two or three different heavy (non-hydrogen) elements (see Fig. 3).

Here, it was found that 68.0% and 65.0% of the QM7-X dataset is comprised of molecular structures which contain only [H,C,N] and [H,C,O] atoms, respectively, while structures containing [H,N,O] atoms are found in 38.7% of the dataset. As such, we consider that QM7-X provides a representative sample of CCS formed by small [C,H,O,N]-based molecules. Regarding S-containing molecules, we still have a considerable number of structures with [H,C,S] (53,833), [H,N,S] (33,936), and [H,O,S] (32,017) combinations (even considering the 13,534 [H,N,O,S]-based molecules in Fig. 3(b)) which might help to describe a wider swath of CCS. On the other hand, only 0.06% of QM7-X includes Cl-containing molecules.

Over 40 different physicochemical properties were computed to describe the structure–property and property–property relationships in QM7-X. In Table 2, we showcase a number of properties obtained from the output of QM calculations performed at the PBE0+MBD level. This QM level of theory has proven to be accurate and reliable for describing intramolecular degrees of freedom in addition to intermolecular interactions in organic molecular dimers, supramolecular complexes, and molecular crystals^41,51,52,56–59^. We therefore consider that these calculations are suitable to validate the quality of future work using this dataset.

**Fig. 4:**
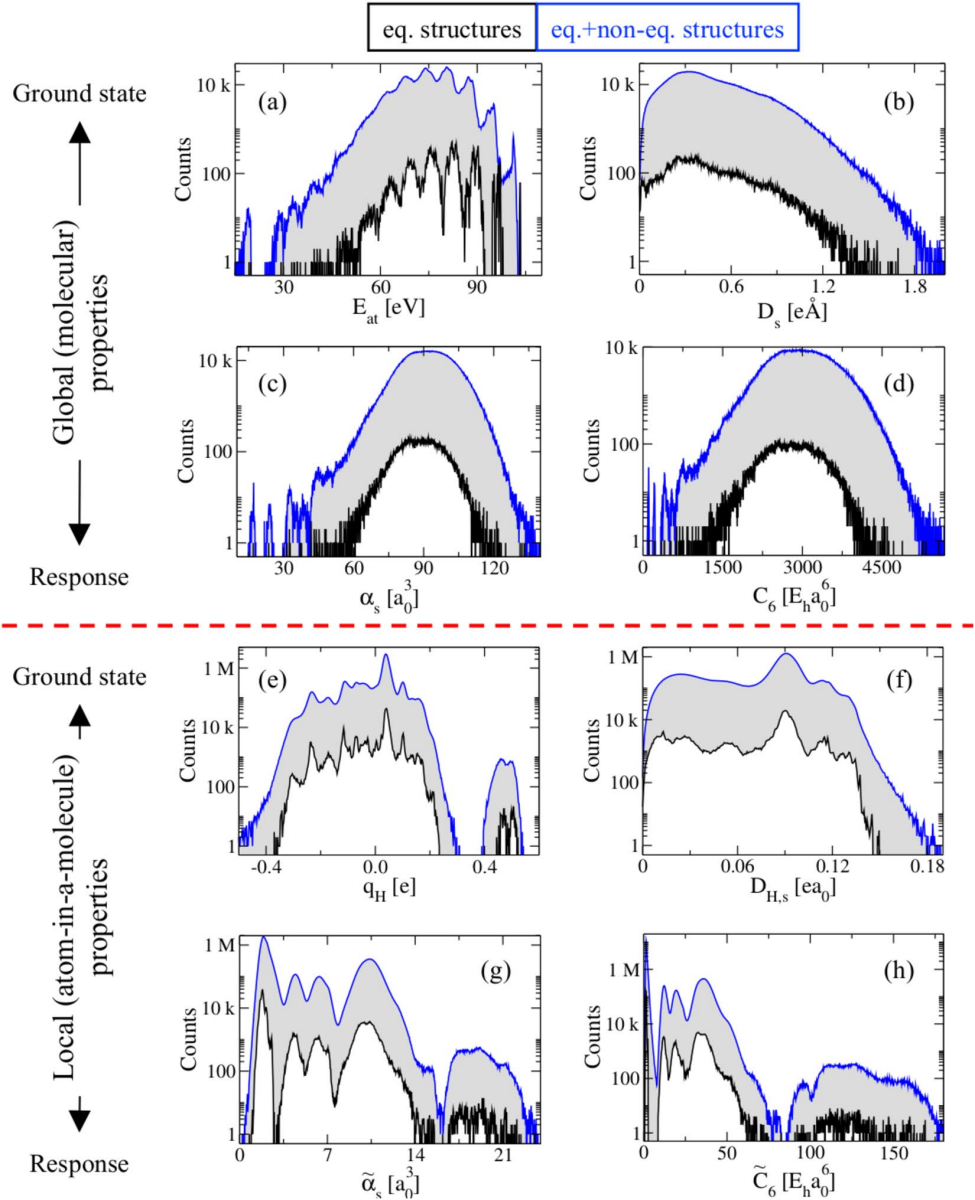
Distribution of physicochemical properties considered in the QM7-X dataset. Global (molecular) properties are considered in the top panel, while local (atom-in-a-molecule) properties are considered in the bottom panel. For each case, we have selected two different examples ground-state and response properties: (**a**) atomization energy, (**b**) scalar dipole moment, (**c**) molecular polarizability (isotropic), (**d**) molecular *C*_6_ coefficient, (**e**) Hirshfeld charges, (**f**) scalar Hirshfeld dipole moment, (**g**) atomic polarizability (isotropic), and (**h**) atomic *C*_6_ coefficient. For each property, we show the results corresponding to the equilibrium structures only (black lines) as well as the entire QM7-X dataset (blue lines). Contributions from non-equilibrium structures are highlighted in grey.

To provide an example of the significant information that can be obtained from QM7-X, we have plotted the distribution of several physicochemical properties in Fig. 4 according to the classification scheme defined in Table 2, *i.e*., division into global (molecular) and local (atom-in-a-molecule) properties, as well as ground-state and response properties. For a cursory look at how some of these properties vary with molecular size, see Fig. S2. Here, the influence of including non-equilibrium structures on a given property is highlighted by comparing the property distributions corresponding to the equilibrium structures only (black lines) with that of the entire QM7-X dataset (blue lines). Overall, one can see many interesting trends in this data. Generally speaking, structural distortions produce significant fluctuations around the values of each property for the equilibrium structures, and therefore improve the exploration and description of molecular property space. In the examples provided here, we find that global properties such as molecular polarizabilities (*α*_*s*_) and dispersion coefficients (*C*_6_) show similar distributions due to the strong correlation existing between them *via* the Casimir-Polder integral^60^ (see Fig. 4(c,d)). Whereas, their local analogs, *i.e*., the atomic polarizabilities (${\widetilde{\alpha }}_{{\rm{s}}}$) and dispersion coefficients (${\widetilde{C}}_{6}$), display characteristic peaks corresponding to the specific local atomic environments found in the equilibrium and non-equilibrium molecular structures (see Fig. 4(g,h)). We also find that intensive properties (*e.g*., HOMO-LUMO gaps and dipole moments) are more sensitive to structural distortions as compared to extensive properties (*e.g*., atomization energies), see Fig. S2. Accordingly, QM7-X offers us the possibility to explore a great diversity of physicochemical properties and to search for unknown correlations among components of the CCS for small molecules. It also opens up a new route for rational design and precise control over the physicochemical properties of small drug-like organic molecules.

Regarding ML applications, we are confident that the data provided by QM7-X will enable continued development of ML approaches, identification of novel QM-based molecular descriptors, as well as determination of robust training sample selection methods that will improve the accuracy and reliability of molecular property predictions. For instance, Stöhr *et al*.^61^ have recently employed different methods for selecting the molecular structures from QM7-X to define training sets for the prediction of DFTB repulsive potentials. In this work, it was found that randomly selected training samples have a better performance compared to more refined training sets (*e.g*., consisting of equilibrium structures together with a given number (*X*) of non-equilibrium structures) when developing accurate many-body repulsive potentials for the DFTB method *via* deep tensor neural networks. Hence, QM7-X can be used to improve the accuracy of electronic structure methods using ML approaches such as Δ-learning. Further improvements of ML models may also be accomplished by considering physics-/chemistry-based approaches for selecting training samples (*e.g*., based on the local chemical environment surrounding each atom).

## Supplementary information

Supplementary Information

## Data Availability

The initial structure generation was carried out by using Open Babel^36^. Further structure optimization and the creation of non-equilibrium structures was performed by utilizing an in-house version of DFTB+ ^47^ together with ASE^48^. Note that all necessary features regarding the utilized DFTB3+MBD approach are available in the current DFTB+ version^62^. All DFT calculations were carried out using FHI-aims^53^ (version 180218).
