# Supplementary material for: QM7-X, a comprehensive dataset of quantum-mechanical properties spanning the chemical space of small organic molecules
Source: Sci Data. 2021 Feb 2;8:43. doi: 10.1038/s41597-021-00812-2 (PMC7854709; doi:10.1038/s41597-021-00812-2)
Supplement: Supplementary file 1 — Supplementary Information [file 41597_2021_812_MOESM1_ESM.pdf]

SUPPLEMENTARY INFORMATION

QM7-X, A comprehensive dataset of quantum-mechanical properties spanning the chemical space of small organic molecules

Johannes Hoja,[1,2] Leonardo Medrano Sandonas,[1] Brian G. Ernst,[3] Alvaro Vazquez-Mayagoitia,[4] Robert A. DiStasio Jr.,*[3] and Alexandre Tkatchenko*[1]

[1] *Department of Physics and Materials Science, University of Luxembourg, L-1511 Luxembourg, Luxembourg.*
[2] *Institute of Chemistry, University of Graz, 8010 Graz, Austria.*
[3] *Department of Chemistry and Chemical Biology, Cornell University, Ithaca, NY 14853, USA.*
[4] *Computational Science Division, Argonne National Laboratory, Lemont, IL 60439, USA.*

**Corresponding authors: Robert A. DiStasio Jr. (distasio@cornell.edu), Alexandre Tkatchenko (alexandre.tkatchenko@uni.lu)*

1 Energy distribution for dataset generation

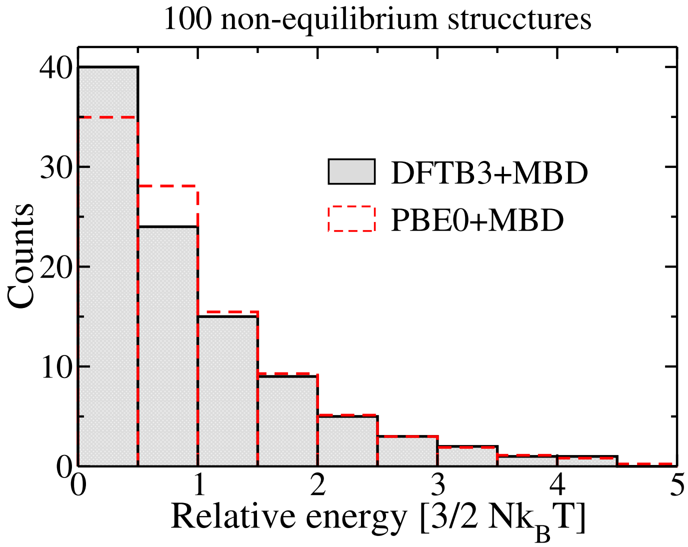

Figure S 1 Energy distribution used to select the non-equilibrium structures for every conformer according to their total DFTB3+MBD energy. For comparison, we have added the resulting average energy distribution calculated with PBE0+MBD.

2 Isomers and conformers information

Chemical formula	Isomers	Conformers
C_5H_9NO	318	1954
$C_4N_2O_8$	295	1812
$C_6H_{10}O$	291	1913
$C_5H_{11}NO$	287	3200
$C_5H_8O_2$	253	1340
$C_4H_6N_2O$	251	630
$C_6H_{11}N$	231	1532
C_6H_8O	225	867
$C_5H_{10}N_2$	219	1583
$C_5H_8N_2$	206	762
$C_6H_{13}N$	197	2040
$C_6H_{12}O$	193	1965
$C_5H_{10}O_2$	188	1881
$C_5H_{12}N_2$	176	2064
C_6H_9N	156	731
$C_5H_6O_2$	130	358
$C_4H_9NO_2$	120	1182
$C_4H_5NO_2$	118	251
C_7H_{10}	108	461
C_7H_{12}	100	685

Table S 1 Number of isomers for the most abundant chemical compounds within the QM7-X database. The "isomers" column enumerates all considered constitutional and stereoisomers (without conformers), while the "conformers" column enumerates the total number of considered conformers.

3 Atomic energies

Chemical element	Energy [eV]
H	-13.641404161
C	-1027.592489146
N	-1484.274819088
O	-2039.734879322
S	-10828.707468187
Cl	-1027.592489146

Table S 2 Atomic energies used to compute atomization energies. These values were obtained at the PBE0 level using `FHI-aims`. Each atom was treated with the proper spin state for the neutral species.

4 Physicochemical properties

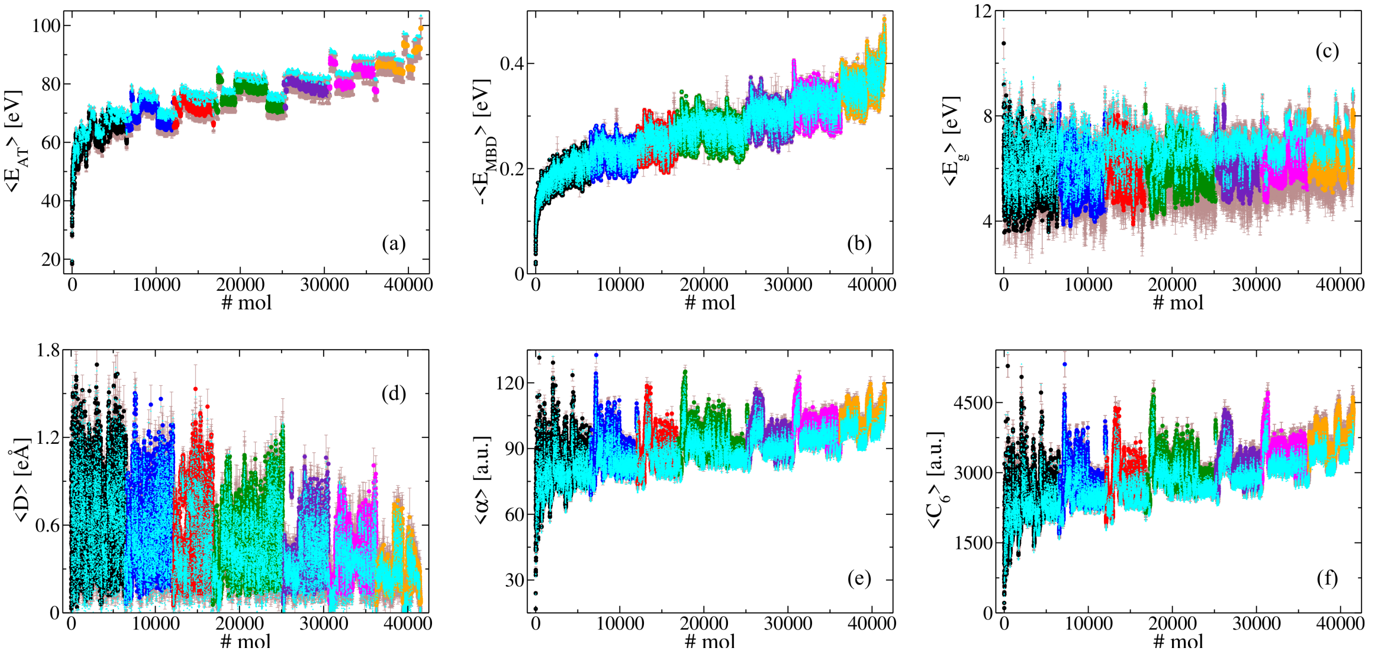

Figure S 2 Variation in physicochemical properties as a function of molecular size (or total number of atoms): (a) atomization energy, (b) MBD dispersion energy, (c) HOMO-LUMO gap, (d) dipole moment, (e) molecular polarizability, and (f) molecular C_6 coefficient. The different colors represent blocks of molecules with different sizes as illustrated in Figure 1 of the main text. The error bar values (brown bars) correspond to twice the value of the standard deviation for a given property for each set of configurations. The corresponding values for the equilibrium structures are shown with cyan △.

5 Energy components

The nuclear-electron attraction E_{ne} and Coulomb energy E_{coul} listed in Table 2 are obtained in the following way from the available energy components of the `FHI-aims` output:

$$E_{\text{ne}} = \text{“Electrostatic energy”} - E_{\text{coul}} - E_{\text{nn}}, \tag{1}$$

$$E_{\text{coul}} = E_{\text{nn}} - \text{“Free-atom electrostatic energy”} - \text{“Hartree energy correction”}, \tag{2}$$

in which E_{nn} is the nuclear-nuclear repulsion. Note that the listed kinetic energy E_{kin} amounts to the kinetic energy output of `FHI-aims` (v180218) plus $6\times$ “Hartree-Fock Energy” to properly account for the amount of exact exchange used in PBE0. This corrected value also corresponds to the kinetic energy output of the current `FHI-aims` version. The total PBE0+MBD energy is then given by

$$E_{\text{tot}} = E_{\text{kin}} + E_{\text{ne}} + E_{\text{coul}} + E_{\text{nn}} + E_{\text{xc}} + E_{\text{MBD}}. \tag{3}$$

6 Conformer Analysis

In order to evaluate the structural agreement between conformers optimized at the DFTB3+MBD and PBE0+MBD levels, we randomly selected the following 10 flexible molecules, each of which has at least five (meta-)stable conformers: m1067-i1, m1927-i3, m2206-i2, m3057-i2, m4012-i1, m4508-i1, m5046-i4, m5451-i1, m6441-i1, m925-i1. For the resulting set of 63 conformers, the RMSD between the DFTB3+MBD- and PBE0+MBD-optimized structures amounted to only 0.1 Å on average. The structural differences are visualized for several conformers in Figs. S3, S4, and S5, where one can see that the DFTB3+MBD-optimized structures are in excellent agreement with those optimized at the PBE0+MBD level. Even for the conformer with the largest observed RMSD (m5046-i4-c3, 0.66 Å), we note that the PBE0+MBD structure did not transform into any of the other considered conformers, and simply resulted from modifications along the backbone structure. As such, this analysis suggests that DFTB3+MBD-optimized structures provide a high-fidelity representation of the critical points on the PBE0+MBD PES.

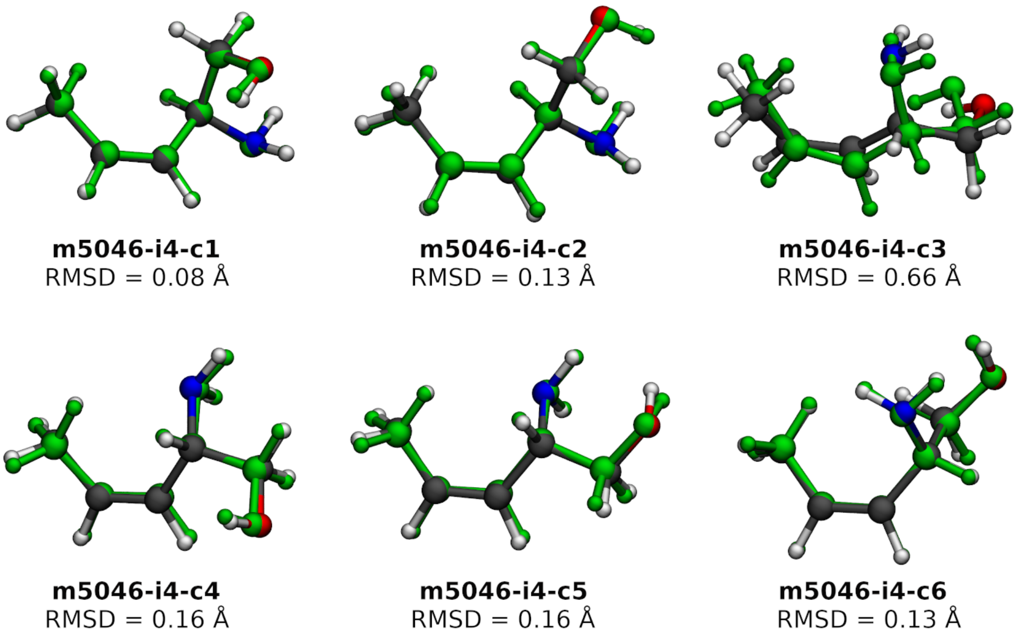

Figure S 3 Comparison between the DFTB3+MBD-optimized structures (full color) and PBE0+MBD-optimized structures (green) for all considered conformers of molecule m5046-i4.

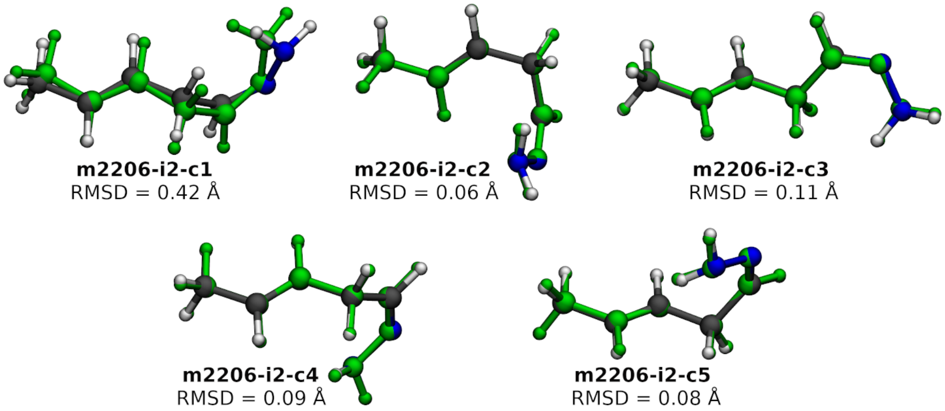

Figure S 4 Comparison between the DFTB3+MBD-optimized structures (full color) and PBE0+MBD-optimized structures (green) for all considered conformers of molecule m2206-i2.

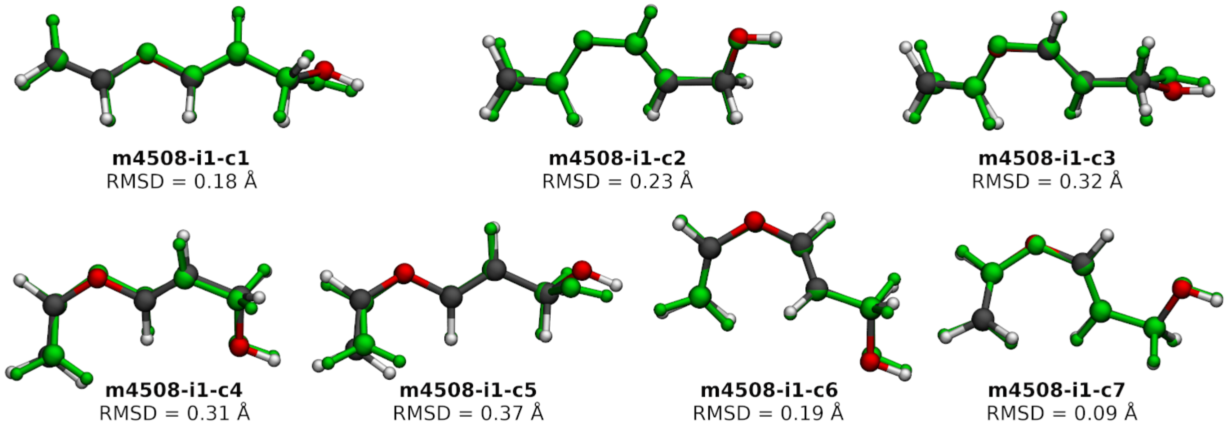

Figure S 5 Comparison between the DFTB3+MBD-optimized structures (full color) and PBE0+MBD-optimized structures (green) for all considered conformers of molecule m4508-i1.
